# Postmarketing experience with Neutrolin® (taurolidine, heparin, calcium citrate) catheter lock solution in hemodialysis patients

**DOI:** 10.1007/s10096-017-3157-7

**Published:** 2017-12-06

**Authors:** Bruce E. Reidenberg, Christoph Wanner, Bruce Polsky, Mariana Castanheira, Alla Shelip, Dirk Stalleicken, Antony E. Pfaffle

**Affiliations:** 1 Department of Pharmacology, Weill Cornell Medicine, 1300 York Ave., New York, NY 10021 USA; 2 Division of Nephrology, University of Wurzburg, Wurzburg, Germany; 3 Department of Medicine, NYU Winthrop Hospital, Mineola, NY USA; 4 JMI Laboratories, North Liberty, IA USA; 5 CorMedix, Inc., 400 Connell Drive, Suite 5000, Berkeley Heights, NJ 07922 USA; 6 CorMedix Europe GmbH, Karlsruhe, Germany

## Abstract

Catheter-related bloodstream infections (CRBSI) are major complications for patients with life-threatening conditions requiring chronic vascular catheterization. The wide range of etiologic microbes and the ongoing development of resistance to antimicrobials with specific mechanisms of action make this an appropriate target for applying a nonspecific antimicrobial therapeutic. Taurolidine hydrolyzes into two antimicrobial moieties, formaldehyde and methylene glycol, which react with microbial surfaces. Neutrolin® (taurolidine, heparin, calcium citrate) was recently introduced in Germany as an antimicrobial catheter lock solution. This postmarketing experience collected data on 201 patients at 20 centers from January 2014 through September 2016. Likely CRBSI was observed in 13 episodes in 47,118 days (0.2759 per 1000 days [0.1468, 0.4718]). Thrombosed catheter was observed in seven catheters in 47,118 days (0.1486 per 1000 days [0.0595, 0.3061]). No adverse drug reactions that led to the discontinuation of Neutrolin® use were reported. Two patients experienced occasional transient dysgeusia. Neutrolin®, when used in conjunction with guideline-based catheter care, showed reduction in the rate of both CRBSI and catheter thrombosis relative to recent historical controls.

## Introduction

Catheter-related bloodstream infections (CRBSI) and thrombosis are the major complications in hemodialysis patients [1]. Microbes known to cause CRBSI include Gram-positive and Gram-negative bacteria, and yeasts [2]. CRBSI are associated with substantial morbidity, mortality, and excess healthcare costs. Patients who receive dialysis through the catheter are 2–3 times more likely to be hospitalized for infection and to die of septic complications than dialysis patients with grafts or fistula [1, 3]. Guideline-based care has had a dramatic impact where implemented. CRBSI rate reduction to 0.5 infections per 1000 patient days was reported in a compliant hemodialysis unit in 2017 [4]. Neutrolin® (CorMedix, Berkeley Heights, NJ, USA) contains taurolidine as its active antimicrobial, which has demonstrated efficacy in children and adults [5, 6]. Taurolidine is a nonspecific antimicrobial with a broad spectrum of activity [7], and no microbial resistance has been observed [8]. Neutrolin® was recently introduced in Germany as an antimicrobial catheter lock solution. Postmarketing experience is important in assessing the CRBSI rate impact of Neutrolin® when used in routine practice.

## Methods

A surveillance program monitoring Neutrolin® use in hemodialysis patients receiving dialysis through tunneled central venous catheters was undertaken at 20 dialysis centers in Germany from January 2014 to September 2016. Hemodialysis patients with new tunneled central venous catheters who had not been hospitalized within the previous 6 months due to CRBSI or catheter thrombosis were selected. Patients received Neutrolin® 2–3 times per week and were followed until either the catheter failed due to clotting or CRBSI, or it is removed due to fistula maturation. Data describing dialysis sessions, likely CRBSI, and catheter thrombi were collected. Investigators reported adverse drug reactions according to postmarketing standards. A total of 201 patients were exposed to more than or equal to one dose of Neutrolin® and they had 15,706 dialysis sessions. Catheter days were estimated by multiplying dialysis sessions by 3. 95% confidence intervals (CIs) of infection rate and thrombosis rate were calculated using Byar’s approximation and the Poisson method, and verified using open source software (http://www.openepi.com).

## Results

The results are displayed in Table 1.

**Table 1:** Postmarketing Neutrolin® experience compared to two historical controls

	Napalkov et al. (2013) [1] Data from 2000–2007	Youssouf et al. (2017) [4] Data from 2010–2011	Neutrolin® Rate [95% CI] Raw data
Infection (likely CRBSI)	4.0/1000 days	0.5/1000 days	0.28 [0.15, 0.47] (13/47,118 days)
Catheter failure Thrombosis	1.26/1000 days	Not collected	0.15 [0.05, 0.31] (7/47,118 days)

### Adverse drug reactions

No adverse drug reactions leading to the discontinuation of Neutrolin® use were reported. Two patients experienced occasional transient dysgeusia.

## Discussion

This paper describes the postmarketing experience with Neutrolin® in dialysis units in Germany after the publication of new guidelines [9, 10] to prevent infection. Though this was an open-label experience, there are two relevant historical controls whose data are displayed in Table 1; Napalkov et al. [1], where the data were collected prior to the institution of these guidelines, and Youssouf et al. [4], where the data were collected afterwards. Comparison of the two historical controls shows a dramatic improvement in infection rates prior to this Neutrolin® experience. The Youssouf et al. [4] experience describes a focused implementation of guidelines with resultant improvement in infection rates. In order to obtain a perspective on whether the low rates observed with Neutrolin® are due to chance alone, 95% CIs were calculated. As can be seen in Table 1, the upper bound of the 95% CI is less than the mean result from Youssouf et al. [4]. Though this cannot be interpreted as a statistically significant difference from historical controls, it is interesting reinforcement of the observed trend. Currently, there is a large, double-blind, randomized, active control study comparing Neutrolin® to heparin in the United States that is statistically powered to deliver definitive results (LOCK-IT-100; see Clinicaltrials.gov). In conclusion, Neutrolin®, when used in conjunction with guideline-based catheter care, can reduce the rate of both CRBSI and catheter thrombosis relative to recent historical controls.
